# Hierarchical Nuclear Architecture in Pre-mRNA Splicing: From IDRs to Speckles and Meshworks

**DOI:** 10.3390/ijms27135954

**Published:** 2026-07-02

**Authors:** Akio Masuda, Tohru Matsuki, Takaaki Okamoto, Naoko Inamura, Masahide Fukada, Yoshiharu Kawaguchi

**Affiliations:** 1 Department of Cellular Pathology, Institute for Developmental Research, Aichi Developmental Disability Center, Kasugai 4800392, Aichi, Japan; matsukit@inst-hsc.jp (T.M.); okamotortho@gmail.com (T.O.); inamura@inst-hsc.jp (N.I.); fukada@inst-hsc.jp (M.F.); kawag001@inst-hsc.jp (Y.K.); 2 Department of Neurobiochemistry, Nagoya University Graduate School of Medicine, Nagoya 4668560, Aichi, Japan

**Keywords:** splicing, RNA-binding protein, speckle, meshwork, intrinsically disordered region, phase separation

## Abstract

The spatial organization of the eukaryotic nucleus plays a pivotal role in regulating pre-mRNA splicing; however, the underlying principles governing this organization remain incompletely understood. Recent advances in imaging and sequencing technologies have revealed that splicing regulation is orchestrated across multiple hierarchical levels, from nanoscale protein–RNA interactions to large-scale nuclear architecture. Intrinsically disordered regions (IDRs) in RNA-binding proteins (RBPs) mediate multivalent interactions that drive liquid–liquid phase separation, leading to the formation of dynamic biomolecular condensates, such as nuclear speckles, paraspeckles, and nuclear stress bodies (nSBs). These structures act as functional hubs that modulate RNA processing efficiency and respond to cellular stress. In addition, emerging evidence highlights nucleus-wide RBP meshworks that spatially organize co-transcriptional splicing through dynamic RNA-dependent interactions. The interplay between these condensates and meshworks forms a spatially organized network that fine-tunes the efficiency and fidelity of pre-mRNA splicing. Collectively, this review presents a unified model in which phase separation and higher-order nuclear architecture coordinately regulate transcriptomic output in space and time.

## 1. Introduction

The eukaryotic cell nucleus is a highly organized structure that serves as the central site for genome maintenance and gene expression. Enclosed by the nuclear envelope, it contains several meters of genomic DNA compacted into a space just a few micrometers in diameter, along with high concentrations of RNA and proteins. These molecules form chromatin, a hierarchically organized structure comprising transcriptionally active euchromatin and repressive heterochromatin, reflecting the dynamic regulation of gene activity. Despite the absence of internal lipid membranes, the nucleus efficiently coordinates numerous complex biochemical processes, including DNA replication, transcription, and RNA processing, with remarkable fidelity and spatiotemporal precision, orchestrating the interactions of thousands of molecules.

Splicing is an indispensable process for mRNA maturation that occurs within the nucleus, facilitating the ligation of exons while excising introns from nascent RNA transcripts. This reaction is catalyzed by the spliceosome, a multi-megadalton ribonucleoprotein complex assembled from five small nuclear ribonucleoproteins (snRNPs) and hundreds of associated proteins, which recognizes target pre-mRNAs with high fidelity and executes each catalytic step with precision [1,2,3]. Furthermore, many RNA-binding proteins (RBPs), including serine/arginine-rich (SR) proteins and heterogeneous nuclear ribonucleoproteins (hnRNPs), participate in splicing regulation [4]. These factors bind to specific exonic or intronic sequence elements in the target pre-mRNA and modulate spliceosome activity by influencing splice site recognition, spliceosome assembly kinetics, and catalytic transitions, thereby playing critical roles in the regulation of alternative splicing.

**Figure 1:**
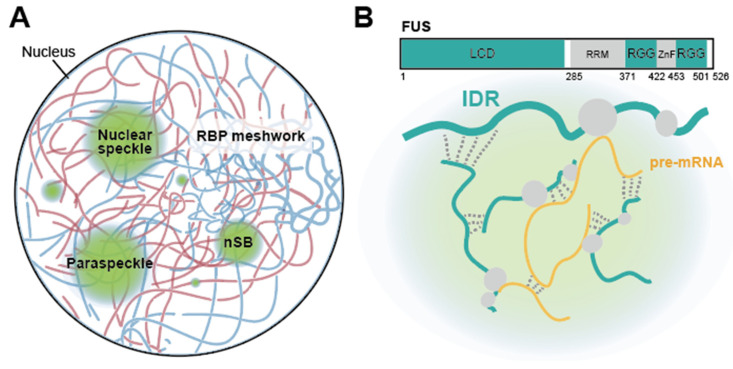
(**A**) Overview of nuclear structures involved in pre-mRNA splicing. Splicing factors are distributed throughout the nucleus, forming RBP meshworks, while also concentrating in discrete nuclear condensates such as nuclear speckles, paraspeckles, and nuclear stress bodies (nSBs). (**B**) (**Top**) Domain organization of FUS as a representative RBP. The low-complexity domain (LCD) and RGG-rich regions (RGG) collectively constitute the IDRs (cyan boxes), whereas the RNA recognition motif (RRM) and zinc-finger motif (ZnF) correspond to structured globular domains (gray boxes). The FUS LCD is enriched in QGSY residues (glutamine, glycine, serine, and tyrosine), while the RGGs contain repetitive RGG (arginine–glycine–glycine) motifs. (**Bottom**) Biomolecular condensates assemble through cooperative and multivalent IDR–IDR, IDR–RNA, and RNA-mediated interactions. IDRs and pre-mRNAs are depicted as cyan and yellow lines, respectively, and gray dashed lines indicate multivalent interactions.

Recent advances in high-throughput sequencing technologies, such as RNA-seq and CLIP-seq, have enabled the systematic mapping of the distribution and interactions of these molecules across the genome and transcriptome. However, these “omic” approaches are inherently limited to a virtual, one-dimensional representation of the genome and thus cannot capture the 3D physical reality of the nucleus. A key question remains: how are splicing factors and their associated RNAs spatially organized within the nucleus to coordinate splicing activity in vivo? Emerging studies using super-resolution microscopy and proximity labeling have revealed that splicing factors and pre-mRNAs concentrate in discrete nuclear bodies and condensates rather than being uniformly distributed. This review explores this organizational hierarchy, from the phase-separating properties of intrinsically disordered regions (IDRs) to nuclear speckles and paraspeckles, and finally to nucleus-wide RBP meshworks that scaffold the splicing process (Figure 1).

## 2. Intrinsically Disordered Regions (IDRs) in Nuclear Organization and RNA Processing

The eukaryotic nucleus contains various membraneless organelles, such as the nucleolus, Cajal bodies, PML bodies, nuclear speckles, paraspeckles, and nuclear stress bodies (nSBs), each performing distinct roles in gene expression. For instance, the nucleolus and Cajal bodies are involved in the biogenesis of ribosomes and snRNPs/snoRNAs, respectively. PML bodies participate in transcriptional regulation and chromatin organization, while nuclear speckles, paraspeckles, and nSBs are associated with transcriptional and post-transcriptional mRNA processing, including splicing [5]. Despite lacking enclosing lipid membranes, each maintains a distinct molecular identity and composition. Early studies focused on discrete molecular interactions, such as protein–protein recognition among splicing factors and snRNP–protein associations, providing important mechanistic insights. More recently, liquid–liquid phase separation (LLPS) has emerged as a complementary framework for understanding the formation of nuclear bodies. In this model, multivalent interactions among biomolecules drive the demixing of specific molecular assemblies from the surrounding nucleoplasm [6]. Among the features that contribute to LLPS, intrinsically disordered regions (IDRs) play important roles, as their flexible and multivalent nature facilitates the weak and transient interactions that underlie condensate formation.

IDRs are functional segments of proteins that lack a fixed three-dimensional structure under physiological conditions (Figure 1B) [7]. Unlike structured globular domains, which often fold into well-defined architectures to perform distinct biochemical functions including enzymatic and binding tasks, IDRs exist as flexible and dynamic conformational ensembles. They are typically characterized by low-complexity sequences enriched in specific residues, such as glycine, serine, arginine, tyrosine, and phenylalanine [8]. The physicochemical properties of these residues enable IDRs to engage in multivalent interactions that drive phase separation. Glycine and serine confer conformational flexibility, allowing IDR chains to sample a wide structural space and maximizing the accessibility of interacting motifs. Arginine supports electrostatic interactions with negatively charged residues and RNA phosphate groups, while also mediating cation–π interactions with aromatic residues [9,10]. Tyrosine and phenylalanine further facilitate π–π stacking and cation–π contacts between adjacent chains [11,12]. Although each individual interaction is weak, their cumulative effect triggers a phase transition once the local concentration of these molecules exceeds a critical threshold. Thus, the compositional bias of IDRs underlies the propensity of IDR-containing proteins to partition into biomolecular condensates [13]. Recently, machine learning-based sequence analyses have identified distinct classes of IDRs that orchestrate phase separation and the spatial organization of functional units [14,15,16,17].

IDRs are frequently found in RNA-binding proteins (RBPs) (Figure 1B) [18]. Among these, SR proteins are characterized by arginine-serine (RS) domains, which mediate protein-protein and protein-RNA interactions critical for spliceosome assembly, often in a phosphorylation-dependent manner [19,20]. Heterogeneous nuclear ribonucleoproteins (hnRNPs) harbor glycine-rich IDRs, which frequently contain arginine-glycine-glycine (RGG) motifs that facilitate RNA binding and promote LLPS [19,21,22]. Additionally, several RBPs, including FUS, TDP-43, and hnRNP A1, contain low-complexity domains (LCDs), often termed prion-like domains, enriched in glutamine, glycine, serine, and tyrosine residues; these domains facilitate their recruitment to biomolecular condensates such as stress granules [23,24,25]. Crucially, disease-associated mutations are frequently found within the IDRs [26]. In particular, mutations linked to Amyotrophic Lateral Sclerosis (ALS) and Frontotemporal Lobar Degeneration (FTLD) are prevalent in the IDRs of RBPs, such as TDP-43, FUS, hnRNP A1, and MATR3 [27]. These mutations have been shown to alter the dynamics of LLPS, promoting a “phase transition” from reversible liquid droplets to pathological solid-like aggregates [28,29,30]. Furthermore, these disruptions significantly impair RNA metabolism, including splicing regulation, which ultimately exerts a profound impact on neuronal differentiation and survival [31,32,33,34].

Several IDRs engage RNA through transient multivalent electrostatic contacts, acting synergistically with canonical RNA-binding domains (RBDs), such as RNA recognition motifs (RRMs) and K-homology (KH) domains, to modulate RNA recruitment into condensates [35,36]. Arginine-rich IDRs interact with RNA via electrostatic interactions with the phosphate backbone and nucleobases [37]. Studies on nuclear speckle-associated proteins have demonstrated that the guanidinium group of arginine mediates multivalent contacts essential for condensate cohesion and speckle incorporation in cooperation with RRMs [38]. Furthermore, it is increasingly recognized that RNA itself possesses intrinsic LLPS capacity, actively contributing to the formation and modulation of biomolecular condensates [39,40].

The functional advantage of these LLPS-driven assemblies lies in their dynamic equilibrium. Within a condensate, individual proteins and RNAs are not immobile; they remain in constant flux, exchanging with the surrounding nucleoplasm on a timescale of seconds. This fluidity is essential for the rapid recruitment and recycling of splicing factors on pre-mRNA [35]. Moreover, by concentrating specific molecules within a confined volume, LLPS facilitates the assembly of multicomponent machineries, such as the spliceosome [41,42]. These principles are particularly well illustrated by nuclear speckles and paraspeckles, where LLPS contributes to their formation and function in pre-mRNA processing [43,44].

## 3. The Functional Landscape of Nuclear Speckles

Nuclear speckles are major subnuclear ribonucleoprotein bodies within the interphase nucleus [45]. For decades, they were considered “storage sheds” for splicing factors [46], but recent studies have revealed their role as dynamic viscoelastic scaffolds that serve as essential hubs for gene expression, particularly in the efficient processing of pre-mRNA.

**Figure 2:**
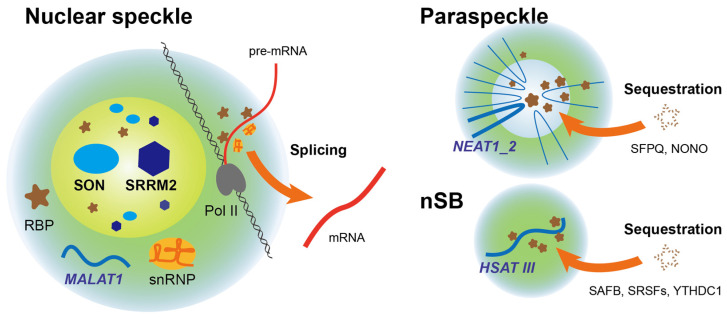
A schematic of nuclear condensates and their functions. Splicing efficiency is enhanced around nuclear speckles by the high concentration of splicing factors, whereas paraspeckles and nSBs sequester specific RBPs to suppress their activity.

Nuclear speckles display a distinct core–shell organization in which SON and SRRM2 localize to the inner core, while the long non-coding RNA (lncRNA) *MALAT1* and various snRNP components are enriched at the periphery, where actively transcribed genes preferentially associate (Figure 2) [47]. SON and SRRM2 are highly disordered proteins that contain extensive IDRs. These proteins act as the primary architectural scaffolds, and their depletion leads to the dissolution of the speckles [48]. Multivalent interactions among these scaffold proteins, particularly SRRM2, confer viscoelastic properties to the speckles [43]. These material properties are dynamically regulated by CK2-mediated phosphorylation of the SRRM2 IDR, which creates alternating charge blocks that intensify intra-network interactions and facilitate speckle relaxation [49]. Recently, microphase separation has been identified as a key mechanism underlying the nanoscale organization of nuclear speckles [50]. RBPs, such as SRSF1 and TDP-43, behave as associative block copolymers in which the interplay of intermolecular attractions and repulsions drives microphase separation, giving rise to size-limited assemblies approximately 25–45 nm in diameter. These microphases further associate with one another to form larger sub-micron-scale clusters observed within speckles. *MALAT1* binds preferentially to SRSF1 microphases to enhance their microphase separation but destabilizes TDP-43 microphases, thereby altering the internal structural organization of nuclear speckles.

Recent studies on genome organization have established that nuclear speckles function as important regulatory hubs that enhance mRNA processing efficiency through spatial organization [51,52,53]. Genes located in proximity to nuclear speckles reside in environments with elevated local concentrations of splicing factors and snRNPs. This facilitates spliceosome engagement with nascent pre-mRNAs and leads to higher levels of co-transcriptional splicing compared with genes positioned farther away in the nucleoplasm. Consistent with a causal role for proximity, the experimental recruitment of pre-mRNAs to nuclear speckles is sufficient to enhance splicing efficiency, indicating that the association with speckles is a key determinant of co-transcriptional RNA processing [51].

The molecular mechanisms underlying this spatial organization involve specific RNA-mediated interactions. A class of nuclear speckle-enriched RNAs containing Alu repeat sequences tethers actively transcribed genomic regions to nuclear speckles through interactions with genomic Alu elements and core speckle proteins [54]. This architectural scaffolding is particularly important during rapid biological transitions, such as erythropoiesis.

Furthermore, the essential role of nuclear speckles in the expression of genes located within GC-rich isochores has been identified quite recently [53]. These genes are enriched in short, GC-rich introns and exhibit a “leveled” exon–intron architecture, in which exons and introns have similar GC compositions, thereby reducing the contrast between splice sites and making their recognition by the spliceosome intrinsically challenging. To facilitate the efficient splicing of these transcripts, nuclear speckles are preferentially positioned in close proximity to these genomic regions. Acute depletion of SON, together with SRRM2, leads to the dissolution of nuclear speckles, which results in splicing defects in a specific subset of GC-rich, speckle-proximal genes, including increased intron retention and exon skipping, while genes outside these regions remain largely unaffected. Consequently, these aberrant GC-rich transcripts are prone to RNA decay pathways, including nonsense-mediated decay (NMD), ultimately leading to marked downregulation of gene expression.

Notably, this study further shows that these GC-rich genes comprise approximately 1500 loci that are typically short, highly expressed, and predominantly associated with essential cellular functions, closely resembling housekeeping genes. Evolutionary analyses indicate that GC-rich isochores and the associated “leveled” exon–intron architecture are largely restricted to amniotes, including mammals and birds [53,55,56]. The expansion of IDRs within SON and SRRM2 coincides with the emergence of these features in the amniote lineage [48]. This phylogenetic distribution suggests that the co-evolution of GC-rich isochores and nuclear speckle-dependent splicing may represent an amniote-specific adaptation, potentially enabling robust and efficient expression of housekeeping genes required to support the increased organismal complexity characteristic of these lineages.

## 4. Paraspeckles: RNA-Scaffolded Sequestration and Stress Response

Paraspeckles are another major class of subnuclear ribonucleoprotein bodies present in the interphase nucleus. Despite their spatial proximity to nuclear speckles, they are immiscible and do not undergo coalescence [57]. Unlike NSs, which facilitate transcription and pre-mRNA processing, paraspeckles act as post-transcriptional regulators of gene expression, particularly during cellular stress [5]. Paraspeckles are organized around lncRNA *NEAT1*, which serves as an essential structural scaffold (Figure 2) [58,59]. *NEAT1* is transcribed as two isoforms: the shorter *NEAT1_1* and the longer *NEAT1_2*, the latter of which is indispensable for paraspeckle assembly [60]. In contrast, *NEAT1_1* localizes to small, discrete nuclear foci distinct from paraspeckles [61], although their functional significance remains to be elucidated [62].

The production of *NEAT1_2* is regulated by alternative 3′-end processing mediated by hnRNP K, which suppresses proximal polyadenylation to promote transcriptional read-through [60]. Upon synthesis, *NEAT1_2* nucleates paraspeckle formation by recruiting various RBPs, including NONO, SFPQ, and PSPC1. These proteins interact with distinct regions of *NEAT1* and drive LLPS through their IDRs. High-resolution imaging has revealed a highly ordered core–shell architecture: the 5′ and 3′ ends of *NEAT1_2* are localized within the shell, whereas the central segments constitute the core, an arrangement that facilitates the selective partitioning of specific RBPs [44].

Functionally, paraspeckles act as dynamic hubs for post-transcriptional regulation. By sequestering specific RBPs, such as SFPQ, within the condensate, paraspeckles reduce their nucleoplasmic availability, thereby modulating downstream transcriptional and post-transcriptional programs [57,63]. Regarding splicing regulation, *NEAT1* has been reported to associate with SRp40 to regulate *PPARγ2* splicing during adipogenesis. Additionally, ARID1B mediates the interaction between paraspeckles and the SWI/SNF complex to co-regulate the alternative splicing of splicing-related genes. Moreover, paraspeckles facilitate the nuclear retention of adenosine-to-inosine (A-to-I) hyperedited RNAs through their interactions with NONO and SFPQ, effectively controlling their cytoplasmic export [64]. *NEAT1* expression is potently induced by various stimuli, such as viral infection [65,66,67,68] and proteotoxic stress [63,69,70], leading to a marked expansion of paraspeckles. This expansion enhances their sequestration capacity, resulting in widespread transcriptomic rewiring and alterations in alternative splicing patterns [71].

Although paraspeckles are largely dispensable for normal development under steady-state conditions [72], *Neat1*-deficient mice exhibit impaired female fertility associated with corpus luteum dysfunction [73], and they are increasingly recognized to play important, context-dependent roles in cellular stress responses [69,70,74]. Accumulating evidence implicates paraspeckle dysfunction in the pathogenesis of various diseases. In oncology, aberrant *NEAT1* expression is frequently associated with tumor progression and poor clinical prognosis [74,75]. In neurodegenerative disorders, dysregulated RNA metabolism and the aggregation of paraspeckle components are thought to contribute to disease mechanisms [76,77]. Additionally, paraspeckles play complex roles in host–virus interactions, where they can either bolster host defenses or be co-opted by viruses to facilitate viral replication [78,79,80].

## 5. Nuclear Stress Bodies (nSBs)

nSBs are specialized subnuclear ribonucleoprotein bodies that primarily form in response to thermal stress [81,82]. These structures nucleate at primate-specific pericentromeric *HSATIII* repeat regions, which remain transcriptionally silent under basal conditions but are rapidly activated by the heat shock transcription factor HSF1 upon stress, leading to the production of *HSATIII* lncRNAs [83]. These lncRNAs serve as essential architectural scaffolds that recruit a diverse proteome to nucleate nSB assembly, including approximately 140 proteins, such as SAFB, HNRNPM, multiple SRSFs, HSF2, Sam68, and CBP (Figure 2) [84,85].

Functionally, nSBs act as regulatory platforms that promote widespread intron retention across hundreds of pre-mRNAs during the post-stress recovery phase through two mechanistically distinct pathways. In the first pathway, nSBs serve as a reaction platform for CLK1-mediated rephosphorylation of sequestered SRSFs, particularly SRSF1 and SRSF9 [85]. Upon stress, SRSFs are globally dephosphorylated. During the recovery phase, CLK1 is recruited to nSBs and rephosphorylates these SRSFs, which in turn promotes intron retention in more than one hundred target transcripts. In the second pathway, nSBs sequester the m6A regulatory machinery [86]. The m6A writer complex is first recruited to nSBs during recovery and methylates *HSATIII* lncRNAs; the resulting m6A-modified *HSATIII* then captures the nuclear m6A reader YTHDC1, depleting it from the nucleoplasm. Since YTHDC1 normally facilitates m6A-dependent splicing of pre-mRNAs, its sequestration within nSBs leads to the retention of a distinct subset of introns. Through these two complementary mechanisms, nSBs orchestrate coordinated transcriptomic reprogramming in response to stress.

## 6. The RBP Meshwork: A Global Architectural Splicing Network

**Figure 3:**
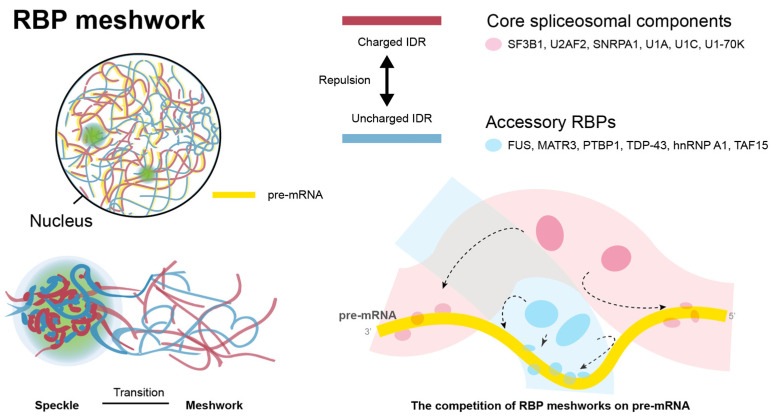
A schematic of the RBP meshwork architecture. (**Top left**) The RBP meshwork consists of two distinct but interconnected networks: one primarily composed of core spliceosomal components harboring charged IDRs (red), and the other composed of accessory RBPs harboring uncharged IDRs (blue). These meshworks are assembled together with pre-mRNAs (yellow), with transcripts preferentially localized within the core spliceosomal meshwork [87]. (**Bottom left**) Meshwork fibers thicken at nuclear speckles. (**Right**) Repulsive interactions between core and accessory RBPs contribute to the formation of two distinct networks, thereby shaping the spatial distribution of RBPs on pre-mRNAs. Arrows indicate the direction of movement of RBPs.

Extending beyond discrete nuclear bodies, recent findings have highlighted a pervasive, nucleus-wide meshwork organization associated with pre-mRNA splicing (Figure 3) [87]. Rather than being uniformly distributed or confined to isolated condensates, multiple splicing-related RBPs assemble into interconnected meshworks that span the nucleoplasm and associate with nascent transcripts.

Decades ago, ultrastructural studies identified the perichromatin region as the primary site of pre-mRNA transcription and co-transcriptional splicing [88]. Perichromatin fibrils arise at the periphery of condensed chromatin and extend into the interchromatin space, forming a reticular network within the nucleoplasm. Harboring nascent pre-mRNA, RNA polymerase II, and spliceosomal snRNPs, these fibrils represent the ultrastructural sites of active transcription and pre-mRNA splicing [89].

Around the same time, a structurally related entity termed “core filaments” was isolated and characterized using electron microscopy [90]. Like perichromatin fibrils, core filaments comprise ~10 nm fibers associated with RNA polymerase II, nascent RNA, and RBPs, and are distributed throughout the nucleus. However, their visualization required extensive sample processing, including high-salt buffer extraction and DNase digestion, raising concerns that these structures might represent preparative artifacts [91]. Consequently, core filaments have largely faded from the literature, although these observations indicate that RNA-RBP complexes can adopt mesh-like organization under certain conditions.

In intact cells, however, in situ cryo-electron tomography did not detect continuous, rigid fiber networks [92], suggesting that nuclear organization is not maintained by stable filamentous scaffolds. Consistent with this, live-cell imaging studies have demonstrated rapid exchange kinetics of splicing factors, indicating that the organization of RNA processing relies on dynamic and weakly organized interaction networks rather than rigid, densely packed structures [93,94]. This dynamic state is supported by abundant nuclear RNA, which modulates RBP interactions and limits irreversible aggregation [95]. These RNA-dependent interactions are largely weak and multivalent, allowing RNA and RBPs to form reversible assemblies that can give rise to molecular networks [96,97,98].

Recent work using super-resolution microscopy extends this model by showing that RNA-dependent, dynamic assemblies can adopt mesh-like organization in cells. SAF-A/hnRNP U forms an RNA-dependent mesh-like organization under physiological conditions, whereas RNA disruption induces rigid aggregates [99,100]. In addition, MATR3, an RBP associated with ALS, interacts with antisense *LINE1* RNAs to form a global meshwork [101]. Various splicing factors have also been shown to assemble into meshworks that span the nucleoplasm [87]. Notably, RNA and RBPs are sufficient to form mesh-like assemblies in vitro, indicating that such mesh-like organization can emerge from weak, multivalent RNA–protein interactions [97]. Relatedly, similar mesh-like organization of mRNAs and RBPs has also been identified in the cytoplasm [102].

These RBP meshwork fibers thicken and densify at nuclear speckles [87], suggesting that nuclear speckles may represent localized, high-density regions within a continuous, nucleus-wide network rather than distinct entities. Consistent with this view, reticular structures can be observed within nuclear speckles [43,57]. Notably, MATR3 forms discrete foci in developing heart and muscle cells in a manner dependent on the expression of a specific lncRNA [103,104], indicating a potential interconversion between localized foci and an extended meshwork state. Together, these findings suggest that local RNA and protein composition likely influences which organizational state predominates, within shared principles of multivalent and RNA-mediated interactions.

The RBP meshworks can be classified into at least two distinct but closely associated networks (Figure 3) [87]. Core spliceosomal components, including SF3B1, U2AF2, SNRPA1, U1A, U1C, and U1-70K, constitute one network, whereas accessory RBPs, such as FUS, MATR3, PTBP1, TDP-43, TAF15, CELF1, and hnRNP A1, form the other adjacent network. Crucially, the maintenance of both RBP meshworks is strictly dependent on active transcription. Inhibition of RNA polymerase II by DRB disrupts the fibrous architecture and transforms it into large spherical droplets, which reversibly reform into filamentous networks upon transcriptional resumption. These observations strongly support the idea that nascent RNA is not merely a passive client within the assemblies, but acts as an essential structural scaffold that spatially templates and stabilizes meshwork organization across the nucleoplasm [87,97].

The molecular basis for the spatial separation of these networks lies in the physicochemical properties of their IDRs. Core spliceosomal components predominantly harbor charged IDRs, whereas accessory RBPs largely carry uncharged IDRs. These distinct classes undergo mutual exclusion during phase separation, thereby preventing their co-assembly into a single network [87]. Similarly, distinct IDR charges play an important role in the separation of transcriptional and RBP condensates [105,106]; greater disparities in IDR charge patterning lead to increased immiscibility, thereby promoting their partitioning into distinct condensed phases. These interactions may be dynamically regulated by post-translational modifications of IDRs, such as phosphorylation, which alters charge properties and can modulate interaction strength and phase behavior [49,107].

This mutual exclusion has functional consequences for RNA processing, as the two meshworks compete for spatial occupancy on pre-mRNA, consequently influencing splicing regulation (Figure 3, right). Consistent with this model, perturbation of meshwork organization alters splicing outcomes independently of overall RBP abundance [87]. Notably, a disease-associated mutation within the IDR of MATR3 modifies this meshwork architecture, leading to aberrant RNA processing. Collectively, these findings suggest that the spatial organization of nucleus-wide RBP meshworks constitutes a critical regulatory layer for alternative splicing that extends beyond the level of individual condensates.

## 7. Conclusions

The spatial organization of the eukaryotic nucleus is increasingly recognized as a dynamic, multi-layered hierarchy driven by the physical principles of biomolecular condensation rather than a static arrangement of isolated compartments. As outlined in this review, the orchestration of pre-mRNA splicing relies on a continuous spectrum of organization, spanning from nanoscale, valence-driven interactions of IDRs to sub-micron non-membranous organelles, such as nuclear speckles, paraspeckles, and nSBs, and ultimately to global, nucleus-wide RBP meshworks.

This physical network provides an essential regulatory layer that optimizes splicing efficiency and shapes transcriptomic outputs. A central challenge in the field is to elucidate how this three-dimensional spatial organization integrates with the dynamic genome architecture over time to precisely regulate RNA metabolism. Addressing this question is critical for establishing a unified framework that links nuclear structure to gene regulatory function in living cells.

## Data Availability

No new data were created or analyzed in this study. Data sharing is not applicable to this article.

## Abbreviations

The following abbreviations are used in this manuscript:

IDR	Intrinsically disordered region
RBP	RNA-binding protein
nSB	Nuclear stress body
snRNP	Small nuclear ribonucleoproteins
SR protein	Serine/arginine-rich protein
hnRNP	Heterogeneous nuclear ribonucleoprotein
LLPS	Liquid–liquid phase separation
RS domain	Arginine-serine domain
RGG motif	Arginine-glycine-glycine motif
LCD	Low-complexity domain
ALS	Amyotrophic Lateral Sclerosis
FTLD	Frontotemporal Lobar Degeneration
RBD	RNA-binding domain
RRM	RNA recognition motif
KH domain	K-homology domain
lncRNA	Long non-coding RNA
